# EGFR/CEP7 high polysomy is separate and distinct from EGFR amplification in glioblastoma as determined by fluorescence in situ hybridization

**DOI:** 10.1093/jnen/nlae028

**Published:** 2024-04-11

**Authors:** Diane M Wilcock, Eric Goold, Lauren M Zuromski, Christian Davidson, Qinwen Mao, Deepika Sirohi

**Affiliations:** Institute for Experimental Pathology, ARUP Laboratories, Salt Lake City, Utah, USA; Institute for Experimental Pathology, ARUP Laboratories, Salt Lake City, Utah, USA; Department of Pathology, University of Utah and ARUP Laboratories, Salt Lake City, Utah, USA; Institute for Experimental Pathology, ARUP Laboratories, Salt Lake City, Utah, USA; Institute for Experimental Pathology, ARUP Laboratories, Salt Lake City, Utah, USA; Department of Pathology, University of Utah and ARUP Laboratories, Salt Lake City, Utah, USA; Institute for Experimental Pathology, ARUP Laboratories, Salt Lake City, Utah, USA; Department of Pathology, University of Utah and ARUP Laboratories, Salt Lake City, Utah, USA; Institute for Experimental Pathology, ARUP Laboratories, Salt Lake City, Utah, USA; Department of Pathology, University of Utah and ARUP Laboratories, Salt Lake City, Utah, USA

**Keywords:** Astrocytoma, Chromosome 7 polysomy, EGFR amplification, EGFR/CEP7 coamplification, EGFR FISH, Glioblastoma, IDH-wildtype glioma

## Abstract

*EGFR* amplification in gliomas is commonly defined by an *EGFR*/CEP7 ratio of ≥2. In testing performed at a major reference laboratory, a small subset of patients had ≥5 copies of both *EGFR* and CEP7 yet were not amplified by the *EGFR*/CEP7 ratio and were designated high polysomy cases. To determine whether these tumors are more closely related to traditionally defined *EGFR*-amplified or nonamplified gliomas, a retrospective search identified 22 out of 1143 (1.9%) gliomas with an average of ≥5 copies/cell of *EGFR* and CEP7 with an *EGFR*/CEP7 ratio of <2 displaying high polysomy. Of these cases, 4 had insufficient clinicopathologic data to include in additional analysis, 15 were glioblastomas, 2 were IDH-mutant astrocytomas, and 1 was a high-grade glial neoplasm, NOS. Next-generation sequencing available on 3 cases demonstrated one with a *TERT* promoter mutation, *TP53* mutations in all cases, and no *EGFR* mutations or amplifications, which most closely matched the nonamplified cases. The median overall survival times were 42.86, 66.07, and 41.14 weeks for amplified, highly polysomic, and nonamplified, respectively, and were not significantly different (p =  0.3410). High chromosome 7 polysomic gliomas are rare but our data suggest that they may be biologically similar to nonamplified gliomas.

## INTRODUCTION

The 2021 CAP guidelines on molecular biomarker testing for the diagnosis of glioblastoma (GBM) uses *EGFR* amplification, *TERT* promoter mutation status, and gain of chromosome 7 with concurrent loss of chromosome10 to help diagnose gliomas that lack necrosis or microvascular proliferation. However, no standardized guidelines exist for determining *EGFR* amplification or gain of chromosome 7 (1). Since copy number gains of chromosome 7 were first described in astrocytic neoplasms (2), the role of trisomy/polysomy of chromosome 7 as it relates to prognosis has been disputed (2–8). Recent advances have demonstrated that chromosome 7 copy number gain is most useful when considered in the context of the genomic landscape of individual tumors (8). Thus, it becomes essential to have well established guidelines on how to designate polysomy 7. Of equal importance is understanding how EGFR amplification occurs and can be accurately quantified, particularly as it relates to polysomy of chromosome 7. A study performed by Bieńkowski et al in 2013 utilized qPCR and fluorescence in situ hybridization (FISH) to establish EGFR amplification and polysomy 7 disparately. They determined that increased *EGFR* amplification was associated with a worse prognosis when in combination with chromosome 7 polysomy as opposed to chromosome 7 polysomy without a relatively increased *EGFR* amplicon (4). Additional studies have provided support that *EGFR* amplification and/or polysomy 7 result in differing prognoses and have highlighted the need to determine whether *EGFR* copy number is increased via gene amplification or polysomy (4–8). The finding that *EGFR* amplification in and of itself has the highest specificity for glioblastoma lends additional support to the need to determine specific cutoffs when using FISH to establish the presence of an *EGFR* amplification versus a chromosome 7 polysomy without *EGFR* amplification (8). Clearly defined criteria for establishing the presence of *EGFR* amplification can help standardize the diagnosis of GBM and may be critically important as additional therapies targeting the *EGFR* tyrosine kinase receptor are developed.

Given this lack of guidelines, a wide range of criteria have been used by different institutions and research studies over the years with the most common amplification criteria utilized being an *EGFR*/CEP7 copy number ratio of ≥2.0 as assessed by FISH (9–17). A recent study compared *EGFR* FISH with real-time quantitative PCR data in an attempt to determine clinical trial inclusion and suggested using a FISH cut-off of *EGFR*/CEP7 ratio of ≥2.0 with at least 50% of nuclei demonstrating *EGFR* amplification. However, they considered tumors with chromosome 7 polysomy without focal amplification of *EGFR* as nonamplified and did not further investigate or address this population (12). *EGFR* has been shown to be amplified in 52% of GBM and in 44% of these cases it is amplified in conjunction with trisomy/polysomy 7 (6). Upon review of the literature, most studies use trisomy and polysomy interchangeably. While CEP7 trisomy is common in gliomas a small population of cases seen at our major reference laboratory display much higher copies numbers (≥5) of both *EGFR* and CEP7 that do not meet the ≥2.0 *EGFR*/CEP7 ratio requirement. While the interchangeability of trisomy and polysomy terminology makes evaluation difficult it appears that this population has not been well studied or characterized in gliomas.

Herein, we sought to evaluate the rare cases with an *EGFR*/CEP7 ratio of <2 but a copy number of ≥5 for both *EGFR* and CEP7 and determine whether a cutoff ratio of ≥2 would correctly classify these tumors according to tumor biology.

## MATERIALS AND METHODS

### Sample selection

A retrospective search of brain samples submitted for *EGFR* FISH performed at a major reference laboratory was performed. A total of 1143 cases were identified. The parametric 95% reference interval upper limit was found to be 5.2 copies of *EGFR* and CEP7 and we rounded down to the closest whole copy number (≥5 copies) and utilized that as our cut-off criteria to capture this highly polysomic outlier population. Statistical analysis was performed on the primary GBM WHO grade 4 highly polysomic cases using an in-house control group of primary GBM WHO grade 4 cases that were submitted for *EGFR* FISH testing over the same time period (n = 307). Patient information, tumor characteristics, molecular test results, and clinical outcome data were collected and analyzed. All patients had a minimum of 1-year follow-up since the time of diagnosis. Survival times were calculated from the day of original biopsy or resection to the death date.

### Fluorescence in situ hybridization

*EGFR* FISH was performed on 3- to 5-µm-thick formalin-fixed, paraffin-embedded (FFPE) tissues. After pretreatment on a Dako PT Link a pepsin digestion step (Agilent Technologies, Santa Clara, CA) was done on a VIP2000. The slides were treated with probes from Abbott Laboratories LSI *EGFR* SpectrumOrange and LSI CEP7 SpectrumGreen probe. The slides were hybridized on a Thermobrite machine (Abbott Molecular, Des Plaines, IL). Following hybridization, slides were washed with Dako Stringency buffer and dehydrated through a series of ethanol solutions. They were counterstained with DAPI and coverslipped. Each FISH slide was scanned manually in its entirety and a minimum of 50 representative tumor nuclei were enumerated on each case by a technologist and reviewed by a pathologist with expertise in solid tumor FISH.

### Immunohistochemistry

IDH1 R132H immunohistochemistry (IHC) staining on tumor tissue sections was performed using the monoclonal mouse H09 antibody clone by Dianova (Geneva, Switzerland) at a 1:200–1:400 concentration on either a Ventana BenchMark Ultra or a Leica Bond Autostainer.

### Molecular studies

Tumor tissue with adequate tumor cellularity, as evaluated by anatomic and molecular oncologic pathologists, was selected for DNA extraction from FFPE tissue sections.

### MGMT methylation

MGMT methylation status was determined by performing a bisulfite conversion on extracted DNA before using PCR amplification and bisulfite-specific primers targeting the MGMT promoter region. Methylation was then detected using either a LightCycler PCR instrument (Roche Diagnostic, Switzerland) or a MassArray MALDI-TOF mass spectrometry (Agena Bioscience, San Diego, CA).

### Mutational analysis

Pyrosequencing of extracted DNA to detect *IDH1* and *IDH2* exon 4 mutations was performed on the Qiagen PyroMark Q24 Pyrosequencer (Qiagen, Germantown, MD).

### Next-generation sequencing

All next-generation sequencing (NGS) testing was performed by accredited outside reference laboratories.

### Statistics

The statistical analysis was performed using R (R Core Team, 2022) (18). Kruskal-Wallis tests were run to compare overall survival and age, while Wilcoxon tests were run for pairwise comparisons of age and sex (adjusted for multiple comparisons using the Holm method).

## RESULTS

**Figure 1.**
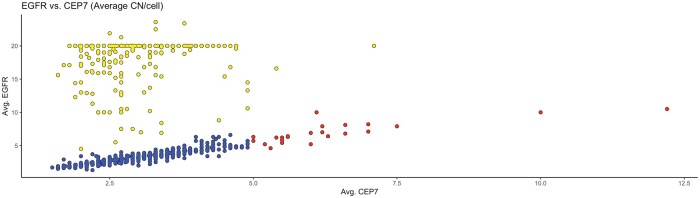
Distribution of *EGFR* and CEP7. Yellow = amplified with *EGFR*/CEP7 ratio ≥2.0, blue = nonamplified with *EGFR*/CEP7 ratio <2.0, red = highly polysomic with *EGFR*/CEP7 ratio <2.0 and average *EGFR* and CEP7 CN ≥5.0.

**Figure 2.**
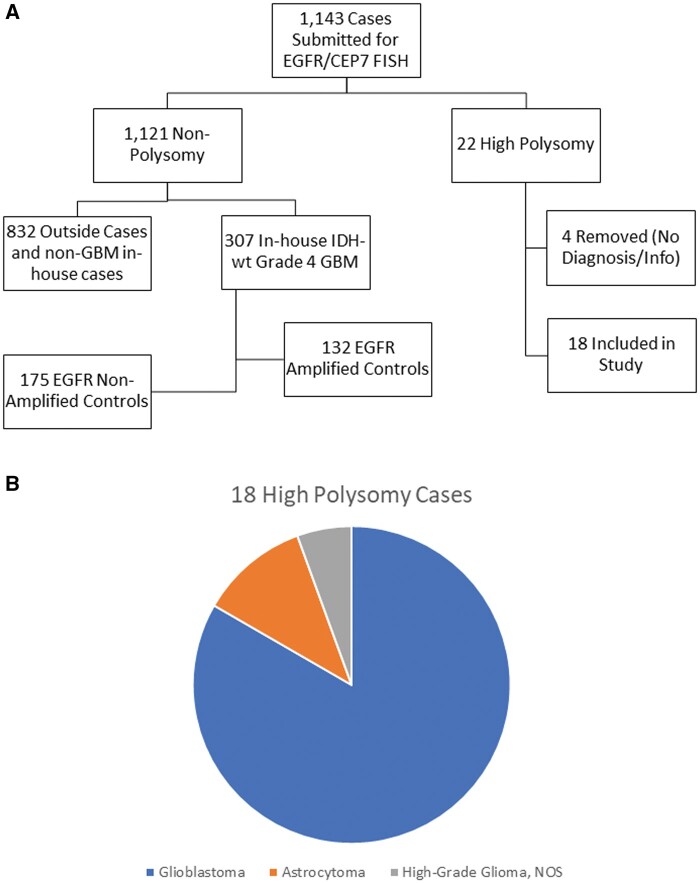
Flowchart of cases. **(A)** All cases represented from initial identification (n = 1143) to those included in the study. **(B)** Pie chart of composition of 18 high polysomy cases included in the study.

With our selection criteria, 1143 cases were identified. The highly polysomic cases comprised 1.9% (22/1143) of the total FISH results. While there were 22 cases that met the highly polysomic criteria; 4 cases had insufficient clinical data for diagnostic classification and were removed from additional analysis. The distribution of the remaining 1139 *EGFR*/CEP7 FISH results is shown in Fig. 1. Of the included 18 cases, 8 cases were from outside clients and 10 were in-house patients; they had been diagnosed as GBM, IDH-wildtype, WHO grade 4 (n = 14), GBM, IDH-wildtype arising from a previously diagnosed low-grade glioneuronal tumor (n = 1), IDH-mutant astrocytoma (n = 2), and a high-grade glial neoplasm, NOS (n = 1). A flowchart of cases is shown in Fig. 2.

### GBM, IDH-wildtype results

#### Control cohort

There was a total of 307 IDH-wildtype glioblastoma, WHO grade 4 control cases that were diagnosed during the same time as our high polysomy GBM cases. This control group was composed of 132 *EGFR*-amplified and 175 *EGFR*-nonamplified cases.

#### Demographics

**Figure 3.**
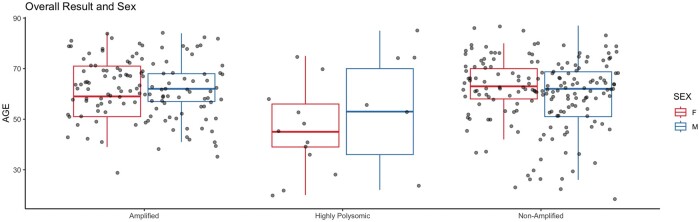
Age and sex by *EGFR* status. Box and whisker plot with female cases in red and male cases in blue.

The median age between the 3 groups of amplified, highly polysomic, and nonamplified were 62, 50.5, and 62 years, respectively. Age was statistically significantly different between both amplified and highly polysomic groups (p = 0.031) and between highly polysomic and nonamplified groups (p = 0.036), but not between the amplified and nonamplified groups (p = 0.906). Highly polysomic and nonamplified cases had a broader range of ages (20–85 and 18–87 years old, respectively), compared to the amplified cases (29–84 years old). There were also no significant differences in age when compared to sex for the 3 populations (Fig. 3).

#### Histopathology

The majority of the highly polysomic GBMs without a prior CNS diagnosis had necrosis (n = 11) and microvascular proliferation (n = 12); however, there were 2 cases (2/14, 14.3%) that lacked both necrosis and microvascular proliferation. One of these 2 cases was reported as a GBM due to the dramatically elevated *EGFR* copy number of 10.5 with concurrent CEP7 copy number of 12.2, thus with a ratio of 0.9. This result was the only finding used to establish the diagnosis of GBM and highlights the difficulty and necessity of establishing more rigorous criteria for *EGFR* amplification. The other case was reported as a GBM from an outside institution; the reasoning for the diagnosis was not provided, perhaps necrosis and/or microvascular proliferation was present on an alternative slide from the one we were sent or one of the other 2 GBM-defining molecular alterations (i.e. *TERT* promoter mutation or a gain in chromosome 7 copy number with concurrent loss of chromosome 10), was known but not provided to our institution.

#### Molecular characteristics

NGS was available on 3 highly polysomic cases, 22 amplified, and 34 nonamplified cases. All 3 of the highly polysomic cases had a *TP53* mutation and only 1 of the 3 cases carried a *TERT* promoter mutation. While the sample sizes were limited, these molecular results more closely aligned with the *EGFR* nonamplified group than the *EGFR* amplified group because *TP53* mutations were seen in only 9% (2/22) of the amplified group but comprised 32.4% (11/34) of the nonamplified group. Conversely, *TERT* promoter mutations were seen in 95.5% (21/22) of the amplified group and only 61.8% (21/34) of the nonamplified group. No *EGFR* amplifications or mutations were detected in the 3 cases with NGS from the high polysomic group, whereas 21/22 (95.5%) and 2/34(5.9%) of the *EGFR* amplified and nonamplified FISH group, respectively, were classified as *EGFR* amplified by NGS. Furthermore, nearly all amplified cases (20/22, 90.9%) also contained an *EGFR* mutation while the nonamplified group only had 3 cases of an *EGFR* mutation (3/34, 8.8%). Two of the 3 nonamplified *EGFR*-mutated cases by FISH correlated with *EGFR*-amplification by NGS. Upon investigation, their *EGFR* nonamplification FISH status had been determined on the initial resection and the NGS had been performed on recurrences/resections 5–6 months later. In that time, it is possible that the tumor developed a detectable population with amplification of *EGFR*.

The mutational burden in the highly polysomic cases was 1.7, 2.5, and 15.8 m/MB. The mutational burden in the amplified and nonamplified groups ranged from 1.0 to 8.0 (mean 4.1) and 0.0 to 34.0 (mean 4.7), respectively. The NGS testing also showed a germline *MYH11* p. A1414T mutation in one highly polysomic patient that would correspond to Marfan/Loeys-Dietz syndrome.

*MGMT* promoter methylation was comparable among all 3 groups with 7/17 (41.2%) high polysomic cases, 45/98 (45.9%) of amplified cases, and 55/141 (39%) of nonamplified cases showing methylation.

**Figure 4.**
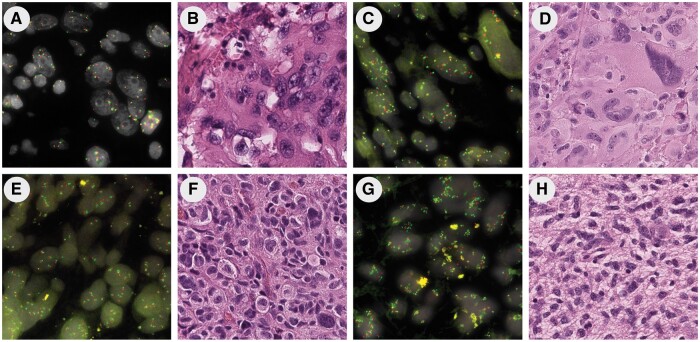
FISH images and corresponding histology in representative tumors. **(A, B)** Case with average of ∼7 *EGFR* CN/cell. **(C, D)** Case with average of ∼8 *EGFR* CN/cell and numerous giant cells. **(E, F)** Case in which the majority of cells showed ∼5 *EGFR* CN/Cell but with ∼5%–10% of cells that had >10 copies of both *EGFR* and *CEP7* and occasional giant cells. **(G, H)** Case with average of >10 *EGFR* and *CEP7* CN/cell. Mag: FISH images, 100×; H&E images, 20×.

Histological features were evaluated in the highly polysomic cases but there were no consistent defining features. However, several cases demonstrated large atypical or giant cell features and/or bizarre multinucleate forms/mitoses (Fig. 4).

#### Survival

**Figure 5.**
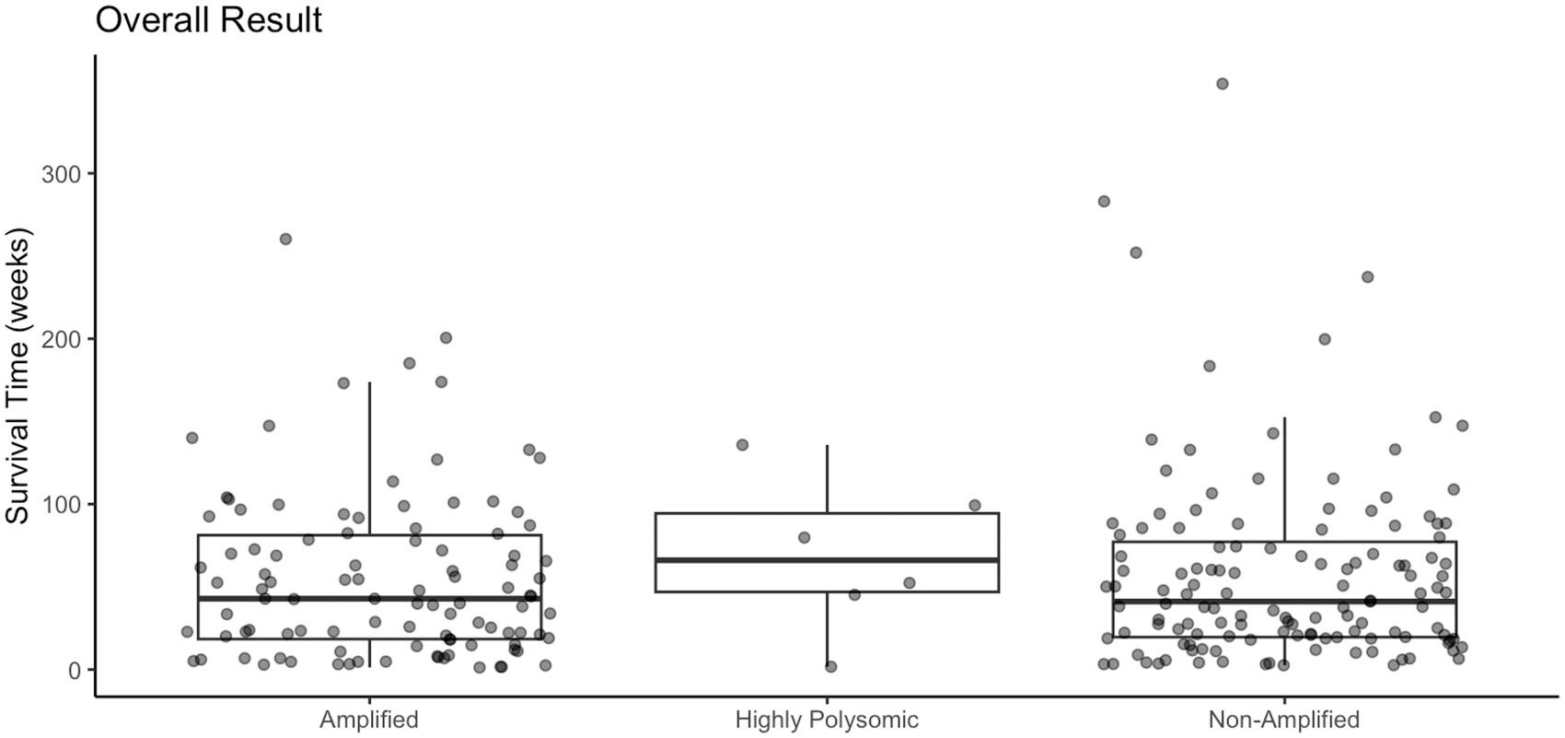
Overall survival by EGFR status. Survival was calculated, in weeks, from date of biopsy/excision to the date of death.

Survival data were available for 102 amplified, 5 highly polysomic, and 131 nonamplified cases (Fig. 5). The median survival was 42.86, 66.07, and 41.14 weeks for the amplified, highly polysomic, and nonamplified groups, respectively. While the median survival was longer for the highly polysomic group, survival times were not significantly different between groups (p = 0.341).

### Glioblastoma, IDH-wildtype arising from a low-grade glioneuronal tumor

One of the highly polysomic cases (1/18, 5.6%) was a GBM that appeared to arise from a low-grade glioneuronal tumor diagnosed 16 years prior to the GBM resection in a patient with neurofibromatosis type 1. The GBM demonstrated microvascular proliferation but no necrosis. Unfortunately, no NGS or additional molecular information was available on this tumor.

### IDH-mutant astrocytoma results

Two of the highly polysomic cases (2/18, 1.1%) were IDH-mutant astrocytomas. IDH1 status was determined using IDH1 R132H IHC. Both cases demonstrated necrosis, although it was focal in one of the cases and that case also showed some features suggestive of a primitive neuronal component. No NGS or additional molecular information was available on these tumors.

## DISCUSSION

Highly polysomic *EGFR* and CEP7 cases as defined above with an *EGFR*/CEP7 ratio of <2 and a copy number of ≥5 for both probes are rare and only comprised ∼1.9% of cases submitted to a major reference lab for *EGFR* FISH testing. Although they demonstrated an elevated copy number of *EGFR*, in our small sample group they appear to be dissimilar to amplified tumors defined by an *EGFR*/CEP7 ratio of ≥2. This is consistent with previously reported studies assessing *EGFR* amplification as it relates to chromosome 7 copy number (4–7). Highly polysomic cases in this study had a better overall median survival than the traditionally amplified group, although this finding was not statistically significant. High polysomic cases were also seen in younger patients (<29 years old), than cases in the amplified group. However, the sample size of the highly polysomic group (n = 18) was small, which may have limited the statistical power to detect significant differences and similarities between the amplified and nonamplified groups.

Molecularly, the highly polysomic cases were more similar to the *EGFR* nonamplified group with all 3 of the NGS-tested cases harboring a *TP53* mutation and only 1 containing a *TERT* promoter mutation. Moreover, *TP53* mutations and *EGFR* overexpression have been previously reported to be mutually exclusive (19). This pattern of mutual exclusivity was also seen in our control samples with only 3.6% (2/56) of our NGS-tested control cases having both a reported *TP53* mutation and *EGFR* amplification.

The fact that high polysomy of *EGFR* and CEP7 was also seen in IDH-mutant astrocytomas, which rarely have an *EGFR* amplification, and a glioblastoma appearing to evolve from a low-grade glioneuronal tumor would seem to further indicate that high polysomy is not synonymous with *EGFR* amplification and that an *EGFR/*CEP7 ratio is more useful for establishing the presence of *EGFR* amplification by FISH testing than solely relying on the *EGFR* copy number.

Although the present data are not conclusive, these findings support distinguishing high polysomy cases from outright *EGFR* amplified cases. We classify high polysomy cases without definitive *EGFR* amplification as those that have copy numbers of *EGFR* and CEP7 ≥ 5 with a ratio of <2. In these cases, we recommend not outright designating a case as positive for *EGFR* amplification but rather as nonamplified or indeterminate with a comment describing that if the diagnosis or treatment depend on the *EGFR* status additional testing such as DNA sequencing, RNA sequencing, and/or expression profiling should be undertaken to establish the amplified or unamplified status of *EGFR.*

Limitations of this study included the small overall sample size of 22 high polysomy cases total with only 3 of these having NGS results available; survival data were also available on only a limited subset of cases. However, *EGFR*-CEP7 high polysomy is extremely rare and thus necessitated including reference laboratory cases, which generally have limited clinical information. Sequencing was not undertaken for our outside cases due to the lack of tissue to perform additional testing, and the tissue being returned to the outside institution; also, there was no availability for patient consent. Additionally, while NGS is worthwhile for looking at mutations across other genes, it can be challenging to ascertain polysomy or *EGFR*/CEP7 ratios for direct comparisons with FISH. Hence, evaluation of publicly available databases, (although these may be fraught with similar issues to using NGS for a direct head-to-head comparison), or additional multicenter studies with a larger subset of highly polysomic *EGFR* and CEP7 patients are needed to establish the exact useful *EGFR/*CEP7 ratio. However, these methods are outside the scope of the present report. The data presented herein support using an *EGFR/*CEP7 ratio to establish the presence of *EGFR* amplification over using the *EGFR* copy number in isolation and distinguishing in clinical reports between *EGFR* amplification and *EGFR-*CEP7 high polysomy.
